# Rind from Purple Mangosteen (*Garcinia mangostana*) Attenuates Diet-Induced Physiological and Metabolic Changes in Obese Rats

**DOI:** 10.3390/nu13020319

**Published:** 2021-01-22

**Authors:** Oliver D. John, Peter Mouatt, Sunil K. Panchal, Lindsay Brown

**Affiliations:** 1 Functional Foods Research Group, University of Southern Queensland, Toowoomba, QLD 4350, Australia; oliverdjohn@outlook.com (O.D.J.); S.Panchal@westernsydney.edu.au (S.K.P.); 2 Southern Cross Plant Science, Southern Cross University, Lismore, NSW 2480, Australia; Peter.Mouatt@scu.edu.au; 3 School of Health and Wellbeing, University of Southern Queensland, Ipswich, QLD 4305, Australia

**Keywords:** *Garcinia mangostana* rind, metabolic syndrome, cardiovascular disease, rats, α-mangostin

## Abstract

The pulp of the purple mangosteen, *Garcinia mangostana*, is a popular tropical fruit but the rind containing xanthones such as α-mangostin together with procyanidins and anthocyanidins is usually discarded as waste. However, this rind has been used in South-East Asia for diarrhoea, dysentery, skin infections and wounds. As xanthones have reported anti-inflammatory and antioxidant responses, this study has determined the bioactive compounds and evaluated the effects of *G. mangostana* rind on physiological, metabolic, liver and cardiovascular parameters in rats with diet-induced metabolic syndrome. Rats fed a diet with increased simple sugars and saturated fats developed obesity, hypertension, increased left ventricular stiffness, dyslipidaemia and fatty liver. Administration of *G. mangostana* rind as 5% of the food to rats with diet-induced metabolic syndrome gave a dose of 168 mg/kg/day α-mangostin, 355 mg/kg/day procyanidins, 3.9 mg/kg/day anthocyanins and 11.8 mg/kg/day hydroxycitric acid for 8 weeks which reduced body weight and attenuated physiological and metabolic changes in rats including decreased abdominal fat deposition, decreased abdominal circumference and whole-body fat mass, improved liver structure and function and improved cardiovascular parameters such as systolic blood pressure, left ventricular stiffness and endothelial function. These responses were associated with decreased infiltration of inflammatory cells, decreased deposition of collagen in both heart and liver and decreased mean adipocyte size in retroperitoneal adipose tissues. We conclude that, in rats with diet-induced metabolic syndrome, chronic intake of *G. mangostana* rind decreased infiltration of inflammatory cells which decreased physiological, metabolic, liver and cardiovascular symptoms.

## 1. Introduction

The genus *Garcinia* is native to Asia, Africa, Australia and Polynesia with more than 300 species in the *Clusiaceae* or *Guttiferae* family [1,2]. In Asia, most *Garcinia* species are distributed in tropical countries including Malaysia, Thailand, Indonesia and the Philippines [2]. *Garcinia* fruits have long been used in the preparation, cooking, presentation and preservation of foods in these Asian countries [2]. While the pulp of fresh mangosteen as the fruit of *Garcinia mangostana* Linn. is sweet, juicy and tangy, the rind (or peel or pericarp) is usually discarded as a waste product [3]. However, the rind has been used as a medicine for hundreds of years, especially in Southeast Asia, for example in diarrhoea, dysentery, skin infections and wounds [4]. The bioactive phytochemicals in mangosteen rind include xanthones such as α-, β- and γ-mangostin [4] with potential health benefits including anti-inflammatory, antioxidant, neuroprotective, cytotoxic and anti-proliferative responses [5,6]. Although reduction of obesity has not been reported in rodent models, *G. mangostana* rind reduced blood glucose and lipid concentrations, blood pressure and liver steatosis, and improved insulin sensitivity in diet-induced obese rats [7,8]. Further, an ethanolic extract of *G. mangostana* rind reduced blood glucose concentrations by increasing the population of insulin-producing β-cells in streptozotocin-induced diabetic rats [9]. The xanthones from *G. mangostana* rind showed anti-inflammatory effects in mice models of inflammation through reduction of tumour necrosis factor (TNF), interleukin (IL)-6, inducible nitric oxide synthase (iNOS) and cyclooxygenase (COX-2) [7].

Metabolic syndrome is a constellation of biochemical, physiological and metabolic parameters such as dyslipidaemia, hypertension, insulin resistance, central obesity and pro-inflammatory state that increases the risk of chronic diseases including type 2 diabetes and cardiovascular disease [10]. The range of health benefits reported for purple mangosteen products may reduce the symptoms of metabolic syndrome in humans by mechanisms including an insulin-sensitising response as with mangostins from fruit pulp in obese females [11] and by decreasing inflammatory markers and increasing antioxidant capacity as with a mangosteen-based beverage [12]. The limited in vivo studies in humans showing weight loss and decreased blood pressure with purple mangosteen-based products as food supplements [13,14] suggest that the compounds found in purple mangosteen rind could be effective in alleviating metabolic syndrome.

This study has characterised the bioactive compounds and then evaluated the effects on the physiological, metabolic, liver and cardiovascular parameters of *G. mangostana* rind in rats fed a high-carbohydrate, high-fat diet which produces changes mimicking human metabolic syndrome [15]. Parameters included body weight, oral glucose tolerance, liver, heart and adipose tissue histology, organ weights including abdominal fat, plasma biochemistry and cardiovascular parameters including systolic blood pressure, left ventricular collagen deposition and stiffness, and isolated thoracic aortic reactivity. Our hypothesis is that consumption of *G. mangostana* rind will improve the chronic physiological, metabolic, liver and cardiovascular changes associated with diet-induced metabolic syndrome in rats.

## 2. Materials and Methods

### 2.1. *Garcinia mangostana* Rind Powder Preparation and Analyses

*G. mangostana* fruits were obtained from Toowoomba local markets supplied from Tully, QLD, Australia. The fruits were then separated into rind, pulp and seed, and weighed. The rind was frozen at −20 °C before freeze-drying and blended into powder. A sample of rind powder was analysed for compound identification and the remaining powder was kept at 4 °C until further use. All solvents and reagents used were high-performance liquid chromatography (HPLC) or analytical grade.

Xanthone analysis was undertaken with a Phenomenex Kinetex C18 HPLC column (100 × 4.6 mm) using a gradient method [16]. Dried ground fruit was quantitatively extracted by mixing 0.25 g powder with 25 mL of acetonitrile in a volumetric flask and sonicated for 30 min. An aliquot of the solvent sample was centrifuged and 200 µL of the supernatant was taken into HPLC vial for analysis. The mobile phases were solvent A (0.1% formic acid, Milli-Q water) and solvent B (acetonitrile with 0.1% formic acid); optimal separations were obtained using a gradient of 60–95% B over 0–28 min, with flow rate of 0.75 mL/minute. Specific detection for each xanthone compound was performed at 240 nm. Reference standard of α-mangostin (Indofine Chemical Company Inc., Hillsborough, NJ, USA) was prepared in acetonitrile at 0.4 mg/mL, then diluted to produce a five-point calibration curve. Quantification was performed based on calibration curve of reference standards, peak area at 240 nm and sample dilution (Supplementary Figure S1). For identification of compounds, the column was linked to an Agilent 6130 single quadrupole mass spectrometer as detector (Agilent Technologies Australia, Mulgrave, VIC, Australia). Total xanthones were calculated as α-mangostin and results were expressed as % *w*/*w*.

Anthocyanin content was measured by chromatography with a Phenomenex Luna C18 HPLC column (250 × 4.6 mm) using a gradient method as described in the British Pharmacopoeia 2016 (BP2016) monograph for analysis of anthocyanin content in bilberry extracts. Samples were prepared in acidic methanol (10 mL), sonicated for 15 min then centrifuged. An aliquot (2 mL) of the supernatant was then diluted with 2 M phosphoric acid up to 10 mL, equilibrated for 15 min then an aliquot taken into a HPLC vial for analysis. The mobile phases were solvent A (8.5% formic acid, Milli-Q water) and solvent B (8.5% formic acid, 22.5% acetonitrile (Scharlau, Chem-Supply, Port Adelaide, SA, Australia), 22.5% methanol, 41.5% water). The gradient started at 7% solvent B, increased to 25% over 35 min, then to 65% solvent B over 10 min, at a flow rate of 1 mL/minute and an injection volume of 10 µL. Specific detection and calibration was performed at 535 nm. Solvents for reference and sample preparation were 2% hydrochloric acid in methanol and 2M phosphoric acid.

The reference standard, cyanidin chloride (Biopurify Phytochemicals Ltd., Chengdu, Sichuan, China; PRF8031044), was prepared in acidified methanol (25 mL), then diluted in 2 M phosphoric acid, 1 mL into 10 mL. Quantification was calculated as described in the BP2016 based on reference standard, peak area at 535 nm and sample dilution. Total anthocyanins were calculated as cyanidin 3-glucoside.

For procyanidins, the analysis was performed with an Agilent 1200 series HPLC, using an Agilent PL1110-6525 PL gel 5 μm 500A 300 × 7.5 mm column. The mobile phase was isocratic 95% tetrahydrofuran and 5% aqueous lithium bromide at 1 mg/mL at a flow rate of 1 mL/minute with detection at 280 nm with Diode-Array Detector. An internal standard solution of the mobile phase with butylated hydroxytoluene at 0.3 mg/mL was used as the solvent for reference standard and sample. The reference standard used for calculating the procyanidin content was procyandin-B_2_ (Biopurify Phytochemicals Ltd., Chengdu, Sichuan, China; PRF7102801) [17].

The analysis of organic acids was based on the 2016 United States Pharmacopeia (USP) *Garcinia* hydroxycitric acid method. The mobile phase was 0.136 % *w*/*v* potassium dihydrogen phosphate (BDH, Product Code 10203.4B 27594) in 3% phosphoric acid (Sigma-Aldrich, Sydney, NSW, Australia; Product Code 04107) adjusted to pH 2.5. Briefly, about 250 mg of the extracts was weighed and extracted in 5 mL of 3% phosphoric acid with sonication for 15 min. After 5 min centrifugation, an aliquot of the supernatant was pipetted in a HPLC vial and run against reference standards of hydroxycitric acid calcium salt (ChromaDex, Los Angeles, CA; Cat No. 00008386), citric acid (Sigma-Aldrich, Cat No. 240621) and malic acid (Sigma, Cat No. 240176). The analysis was performed using a Phenomenex 250 mm C18 column with 1 mL/minute of isocratic mobile phase over 25 min. Reference standards were injected and prepared as a calibration curve for calculation of the hydroxycitric and citric acid concentrations. For the hydroxycitric acid measurements, the rind of *Garcinia quaesita* was held as a reference sample at Analytical Research Laboratory, Southern Cross University, Lismore, NSW, Australia for herbal authentication [18].

### 2.2. Rats and Diets

The experimental group consisted of 48 male Wistar rats (8–9 weeks old) obtained from the Animal Resource Centre, Murdoch, WA, Australia. Rats were housed individually in a temperature-controlled room (22 ± 2 °C) under 12-h light/dark cycle environment with unrestricted access to food and water at the University of Southern Queensland animal house.

Rats were acclimatised for one week. Upon reaching 335–340 g body weight, they were randomly divided into 4 experimental diet groups (n = 12 per group). Two groups were fed either corn starch (C) or high-carbohydrate, high-fat (H) diets for the full 16 weeks. The other two groups received C or H diets for eight weeks and then received *5% G. mangostana* rind powder added to these diets for the final eight weeks (CM and HM, respectively). The C diet contained 570 g of corn starch, 155 g of powdered rat food (Specialty Feeds, Glen Forest, WA, Australia), 25 g of Hubble, Mendel and Wakeman salt mixture (MP Biomedicals, Seven Hills, NSW, Australia), and 250 g of water per kilogram of diet. The H diet contained 175 g of fructose, 395 g of sweetened condensed milk, 200 g of beef tallow, 155 g of powdered rat food (all obtained from local food suppliers and supermarkets), 25 g of Hubble, Mendel and Wakeman salt mixture and 50 g of water per kilogram of diet. In addition, the drinking water for the H and HM groups was supplemented with 25% fructose [15].

Body weight, food and water intakes measurements were conducted daily and feed efficiency was calculated [15]. Daily energy intake was calculated from the daily water and food intakes during the last 8 weeks of protocol [15]. The difference in body weight between week 8 and week 16 was recorded as increase in body weight.

### 2.3. Rat Measurements

Oral glucose tolerance tests were performed on rats after overnight (12-h) food deprivation; the fructose water in H and HM groups was replaced with normal water. Basal blood glucose concentrations were measured in blood collected from the tail vein after food deprivation and analysed using glucometer (Freestyle lite, Abbott Diabetes Care, VIC, Australia). The rats were then given 2 g/kg glucose through aqueous glucose solution via oral gavage and blood glucose measurements were performed at 30, 60, 90 and 120 min after glucose loading using tail vein prick method [15].

Systolic blood pressure was measured at 8 and 16 weeks with rats lightly sedated under isoflurane gaseous anaesthesia (Lyppard Australia Ltd. Pty, Northgate, QLD, Australia). Measurements were performed using an MLT1010 Piezo-Electric Pulse Transducer (ADInstruments, Bella Vista, NSW, Australia) and an inflatable tail-cuff connected to an MLT844 Physiological Pressure Transducer (ADInstruments) connected to a PowerLab data acquisition unit (ADInstruments) [15].

Dual-energy X-ray absorptiometry (DXA) was performed on rats at the end of the protocol using a Norland XR46 DXA instrument (Norland Corp., Fort Atkinson, WI, USA) [15]. Rats were anaesthetised by isoflurane gaseous anaesthesia and under constant monitoring. Indirect calorimetry was used to study oxygen consumption and carbon dioxide production with a 4-chamber OxyMax system (Columbus Instruments, Columbus, OH), keeping one rat per chamber [19]. Rats had free access to water and food during the experiment. Carbon dioxide production (V_CO2_) and oxygen consumption (V_O2_) were obtained from each chamber. Respiratory exchange ratio (RER = V_CO2_/V_O2_) was calculated by OxyMax software (v. 4.86); fatty acids and carbohydrate oxidation produce RER values of 0.70 and 1.00, respectively. Energy expenditure was calculated using the exchange of oxygen for carbon dioxide that takes place during food metabolism.

The isolated Langendorff heart preparation was performed to assess left ventricular function of the rats in all groups [15]. Lethabarb (pentobarbitone sodium, 100 mg/kg; Virbac, Peakhurst, NSW, Australia) administered intraperitoneally was used to induce terminal euthanasia. Following heparin (Sigma-Aldrich Australia, Sydney, NSW, Australia) administration (~200 IU) into the right femoral vein, blood (~5 mL) was collected from the abdominal aorta in heparinised tubes. Isovolumetric ventricular function was measured by inserting a latex balloon catheter into the left ventricle of the isolated heart connected to a Capto SP844 MLT844 physiological pressure and Chart software on a MacLab system (ADInstruments) [15]. The right and left ventricles were separated after perfusion experiments and weighed. Following removal of the heart, the liver, and retroperitoneal, epididymal and omental fat pads were collected and blotted for weighing. Retroperitoneal, epididymal and omental fat pads were calculated together as total abdominal fat. Organ weights were normalised relative to the tibial length at the time of their removal (in mg/mm).

For measurement of isolated tissue reactivity, approximately 4 mm sections of thoracic aorta were suspended in an organ bath containing Tyrode physiological salt solution bubbled with 95% O_2_–5% CO_2_ and allowed to stabilise at a resting tension of approximately 10 mN. Cumulative concentration-response curves (contraction) were plotted for noradrenaline and cumulative concentration-response curves (relaxation) were plotted for sodium nitroprusside and acetylcholine after submaximal (~70%) contraction with noradrenaline [15].

Approximately 5–7 min after euthanasia, heart, ileum, colon and liver portions were collected and fixed in 10% neutral buffered formalin for 3 days. The samples were then dehydrated and embedded in paraffin wax. Thin sections (~5 µm) of left ventricle, ileum, colon and liver were cut and stained with haematoxylin and eosin. Stained sections were examined using an EVOS FL Colour Imaging System (v 1.4 (Rev 26059); Advanced Microscopy Group, Bothell, WA) to observe infiltration of inflammatory cells in liver and heart, for determining fat vacuoles in liver and to observe gut structural changes [15]. Heart sections were also stained with picrosirius red staining to study collagen distribution in the left ventricle using BX53 Olympus microscope [15].

About 0.5 cm^3^ of retroperitoneal adipose tissue was fixed in 10% neutral buffered formalin and kept in 4 °C for at least 72 h. After that, the formalin was discarded and replaced with 70% ethanol solution. Tissue sections about 10 µm thick were stained with haematoxylin and eosin stain [20]. Sectioned slides were observed using EVOS FL Colour Imaging System (v 1.4 (Rev 26059); Advanced Microscopy Group) to observe deposition of fat. Mean adipocyte area and area distribution were calculated using ImageJ software [21].

Small portions of liver were embedded in Tissue-Tek O.C.T. Compound (ProSciTech, Kirwan, QLD, Australia) and stored at −20 °C. Tissue was sectioned using a cryostat machine (10 µm), dried and stained using Oil red O stain. Stained sections were observed using EVOS FL Colour Imaging System to observe deposition of fat. Mean areas of fat vacuoles were calculated using Image J software [21].

Blood was centrifuged at 5000× *g* for 10 min within 30 min of collection into heparinised tubes. Plasma was stored at −20 °C before analysis. Plasma concentrations of total cholesterol, triglycerides and non-esterified fatty acids, and activities of plasma alanine transaminase and aspartate transaminase were determined using kits and standards supplied by Olympus (Tokyo, Japan) using an AU 400 Olympus analyser [15].

Liver glycogen and plasma catalase activity were measured using published methods with modifications [22,23]. Faecal samples were collected in vivo during euthanasia to measure faecal lipid content using the Folch method [24]. In brief, 1 g of faeces were air-dried and homogenised in 5 mL saline solution. The homogenisation process produced a suspension that was mixed with 5 mL of 2:1 (*v*/*v*) chloroform:methanol mixture and centrifuged at 1000× *g* for 10 min. After centrifugation, the lower liquid phase was separated and transferred into a pre-weighed test tube. The lipids were air-dried and weighed.

### 2.4. Statistical Analysis

All data are presented as mean ± standard error of the mean (SEM). Physiological data were tested for variance using Bartlett’s test and variables that were not normally distributed were transformed (using log10) prior to statistical analyses. The effects of diet, treatment and their interactions on physiological variables were assessed by two-way analysis of variance (ANOVA). When interaction and/or the main effects were significant, means were compared using Newman-Keuls multiple comparison *post hoc* test. *p* value of <0.05 was considered as statistically significant. All statistical analyses were performed using Prism version 6.00 for Windows (GraphPad Software, San Diego, CA, USA).

## 3. Results

### 3.1. Weight and Phytochemical Analysis of Garcinia mangostana Rind

**Figure 1:**
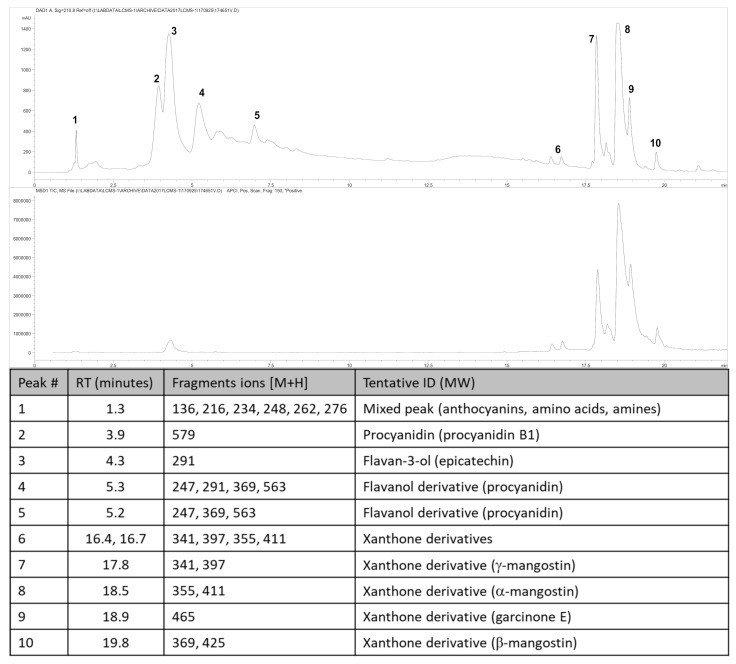
High-performance liquid chromatography (HPLC) chromatogram and mass spectrometric analysis from *Garcinia mangostana* rind for phytochemical analysis at 210 nm (top chromatogram) and xanthone analysis at 240 nm (bottom chromatogram), and peak identification of chromatograms; RT, retention time; ID, identification. Further description of the characterisation and quantification of compounds can be found in the Supplementary Material.

The fruit weighed 95.3 ± 3.2 g (n = 6). Wet weights of the rind, pulp and seed were 69.8 ± 3.2 g, 22.7 ± 2.2 g and 3.8 ± 0.4 g, respectively. The loss upon drying of the rind, pulp and seed was 65%, 85% and 30%, respectively. The total xanthone content of the rind was 6.49% *w*/*w* with α-mangostin (5.42% *w*/*w*) as the major compound with smaller amounts of γ-mangostin, β-mangostin and garcinone E (Figure 1). The total content of procyanidins calculated as procyanidin B_2_ was 12.6% *w*/*w*, the total anthocyanins content in the rind, calculated as cyanidin glycoside, was 0.137% *w*/*w* (cyanidin chloride (0.095% *w*/*w*)) and the total hydroxycitric acid content was 0.42% *w*/*w*.

### 3.2. Metabolic Parameters

**Figure 2:**
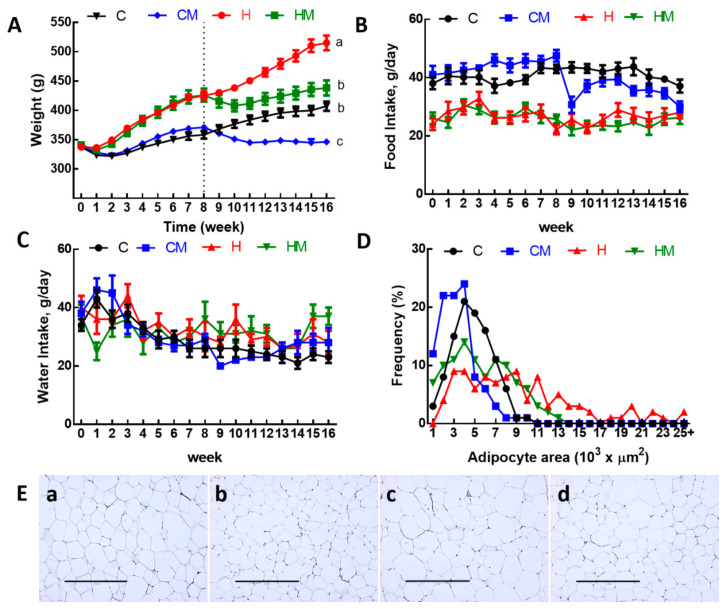
(**A**) Weekly body weight, (**B**), food intake, (**C**) water intake, (**D**) distribution of adipocyte area and (**E**) haematoxylin and eosin staining of adipocytes (magnification ×10; scale bar = 400 µm) in C, CM, H and HM rats; C, corn starch diet-fed rats; CM, corn starch diet-fed rats supplemented with *Garcinia mangostana* rind; H, high-carbohydrate, high-fat diet-fed rats; HM, high-carbohydrate, high-fat diet-fed rats supplemented with *Garcinia mangostana* rind.

Body weights were increased in H rats compared to C rats (Table 1, Figure 2A). Food intake (Figure 2B) was higher in C rats than in H rats, but water intake was not different (Figure 2C). Due to the higher energy content in H diet including fructose in the drinking water, the energy intakes in H rats were higher than in C rats (Table 1). Compared to C rats, H diet for 16 weeks increased total body fat mass, abdominal fat pads and abdominal circumference (Table 1).

After the first 8 weeks of the protocol, there was no difference in lean mass between C and H rats but higher fat mass in H rats than in C rats (Table 1). For the final 8 weeks, the consumption of food in CM rats was higher than in HM rats, leading to the higher dose of α-mangostin in CM rats compared to HM rats (Table 1). Treatments did not reduce food and water intake in either CM or HM rats except in week 9 for CM rats (Table 1, Figure 2B,C). At 16 weeks following 8 weeks of treatment, both CM and HM rats had lower body weight, energy intake, feed conversion efficiency, body weight gain, abdominal circumference, fat mass, retroperitoneal fat, epididymal fat, omental fat, total abdominal fat and visceral adiposity index compared to C and H rats, respectively (Table 1, Figure 2A). Adipose tissue histological analysis showed a reduction of mean adipocyte area in HM rats compared to H rats and in CM rats compared to C rats (Figure 2D). The distribution of the adipocyte area showed more cells with larger area in H rats compared to HM, C or CM rats (Figure 2E).

Metabolic chamber analysis showed unchanged mean and area under the curve for RER between C and CM and H and HM rats. Similarly, there was no difference between mean heat generated between C and CM rats and H and HM rats. However, area under the curve analysis for heat showed decreases in both CM and HM rats, compared to C and H rats, respectively (Table 1).

Plasma analyses showed no difference in aspartate transaminase activity between C and CM rats, but HM rats had reduced activity compared to H rats. There was increased plasma alanine transaminase activity in CM and HM rats compared to C and H rats, respectively. The total cholesterol concentration was reduced in CM rats compared to C rats, but there was no difference between H and HM rats. The plasma triglyceride concentration was increased in H rats compared to C rats but similar between C and CM rats and between H and HM rats. Concentrations of non-esterified fatty acids were decreased in HM rats compared to H rats, but no difference was observed between C and CM rats (Table 1).

At 8 weeks, there was no difference in oral glucose response between C and CM rats and H and HM rats (Table 1). Rats in H groups had higher area under the curve for glucose response than rats in C groups indicating the development of glucose intolerance. At 16 weeks, HM rats showed improvement in oral glucose response while CM rats did not show any difference from C rats (Table 1). Plasma catalase activity was lower in C rats compared to H rats (Table 1). Faecal lipids showed no difference in excretion between H and HM rats or between C and CM rats (Table 1).

### 3.3. Liver and Gastrointestinal Structure and Function

**Figure 3:**
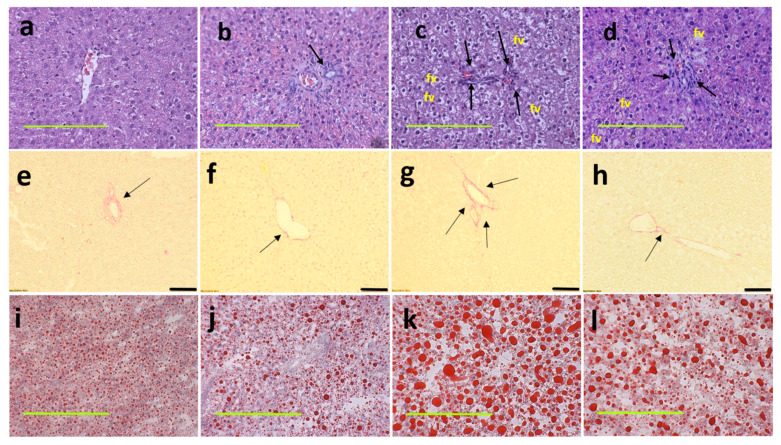
Haematoxylin and eosin staining of liver sections indicating inflammatory cells as dark spots (marked by arrow) and fat vacuoles (marked as “fv”) (**a**–**d**; magnification ×20; scale bar = 200 µm); picrosirius red staining indicating collagen deposition as red stain around blood vessels (marked by arrows) (**e**–**h**; magnification ×12.6; scale bar = 100 µm); Oil red O stain showing fat droplets in red (**i**–**l** magnification ×20; scale bar = 200 µm) in corn starch diet-fed rats (**a**,**e**,**i**); corn starch diet-fed rats supplemented with *Garcinia mangostana* rind (**b**,**f**,**j**); high-carbohydrate, high-fat diet-fed rats (**c**,**g**,**k**); and high-carbohydrate, high-fat diet-fed rats supplemented with *Garcinia mangostana* rind (**d**,**h**,**l**).

**Figure 4:**
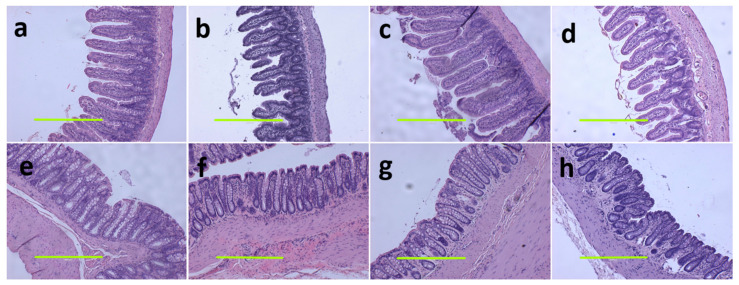
Haematoxylin and eosin staining on ileum (**a**–**d**) and colon (**e**–**h**) (magnification scale bar = 400 µm) in corn starch diet-fed rats (**a**,**e**); corn starch diet-fed rats supplemented with *Garcinia mangostana* rind (**b**,**f**); high-carbohydrate, high-fat diet-fed rats (**c**,**g**); and high-carbohydrate, high-fat diet-fed rats supplemented with *Garcinia mangostana* rind (**d**,**h**).

At 16 weeks, histological analysis of the liver showed increased fat vacuoles in H rats but reduced fat vacuoles in HM, and few vacuoles in C and CM rats (Figure 3a–d). Decreased infiltration of inflammatory cells was observed in HM rats compared to H rats (Figure 3c,d). Mean fat vacuole area was the highest in H rats followed by HM rats, CM and C rats (Table 1). Further, reduced collagen deposition was observed in HM rats compared to H rats (Figure 3g,h). Liver glycogen was reduced in HM rats but not in H, C or CM rats (Table 1). There was no difference in wet liver weight between C and CM and between H and HM rats but liver wet weight was increased in H rats compared to C rats. *G. mangostana* rind treatment did not change colon or ileum structure compared to controls (Figure 4).

### 3.4. Cardiovascular Structure and Function

**Figure 5:**
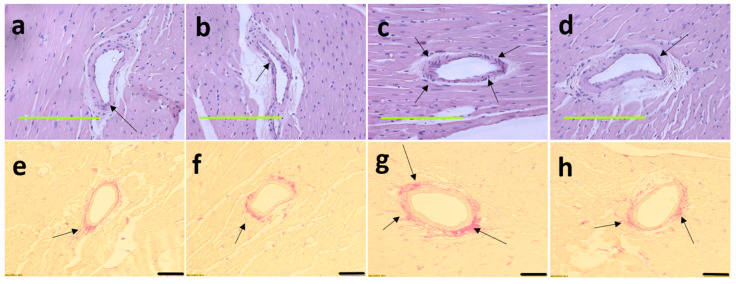
Haematoxylin and eosin staining on left ventricles indicating inflammatory cells as dark spots outside myocytes and in cardiac endothelium tissue (marked as “in”) (**a**–**d**; magnification ×20; scale bar = 200 µm) and picrosirius red staining on left ventricles indicating collagen deposition as red stain around cardiac endothelium tissue (marked by arrows) (**e**–**h**; magnification ×12.6; scale bar = 100 µm) in corn starch diet-fed rats (**a**,**e**); corn starch diet-fed rats supplemented with *Garcinia mangostana* rind (**b**,**f**); high-carbohydrate, high-fat diet-fed rats (**c**,**g**); and high-carbohydrate, high-fat diet-fed rats supplemented with *Garcinia mangostana* rind (**d**,**h**).

**Figure 6:**
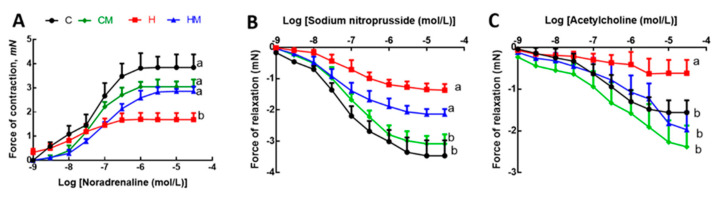
Effects of *Garcinia mangostana* rind on responses of thoracic aortic rings to noradrenaline (**A**), sodium nitroprusside (**B**) and acetylcholine (**C**). Values are shown as mean ± SEM, n = 8–10. Groups with different letters are significantly different, *p* < 0.05. C, corn starch diet-fed rats; CM, corn starch diet-fed rats supplemented with *Garcinia mangostana* rind; H, high-carbohydrate, high-fat diet-fed rats; HM, high-carbohydrate, high-fat diet-fed rats supplemented with *Garcinia mangostana* rind.

After 8 weeks on high-carbohydrate, high-fat diet, systolic blood pressure was increased in H rats compared to C rats (Table 1). At 16 weeks, this increase in H rats was sustained but HM rats showed reduced systolic blood pressure (Table 1). There was no difference in systolic blood pressure between C and CM rats (Table 1). Left ventricular diastolic stiffness was lower in HM rats than in H rats and similar to C rats (Table 1). Infiltration of inflammatory cells and perivascular collagen deposition were increased in the left ventricles of H rats compared to HM rats (Figure 5). There was reduced infiltration of inflammatory cells and collagen deposition in C and CM rats (Figure 5). Noradrenaline-induced thoracic aortic contraction was decreased in H rats compared to C rats; responses were increased in HM rats and were not different from C and CM rats (Figure 6). Sodium nitroprusside-induced relaxation showed no difference in response between H and HM or C and CM rats (Figure 6). Acetylcholine-induced relaxation showed increased responses in C, CM and HM rats compared to H rats (Figure 6).

## 4. Discussion

*G. mangostana* rind contained α-mangostin, procyanidin B1, anthocyanins and hydroxycitric acid. The most likely anthocyanins are cyanidin glucoside and sophoroside based on the chromatographic similarity to published analyses [25]. This combination of phytochemicals decreased the physiological, metabolic, liver and cardiovascular changes in diet-induced metabolic syndrome in rats. The rind showed anti-obesity effects as decreases in body weight, abdominal fat pads, mean adipose cell size and abdominal circumference in high-carbohydrate, high-fat diet-fed rats. The rind reduced liver fat vacuole size, infiltration of inflammatory cells and perivascular collagen deposition. Further, the rind also decreased blood pressure, and reduced infiltration of inflammatory cells and collagen deposition in the left ventricle.

The rind of *G. mangostana* is often regarded as waste but mangosteen peel accounts for over 70% of the whole fruit weight, typical for tropical fruits [26]; the study of health properties may value-add to this abundant waste product. We suggest here that this rind is a valuable resource with potential therapeutic effects in metabolic syndrome. We have likewise reported that the rind of *G. humilis* fruit (achacha) containing procyanidins showed cardioprotection in metabolic syndrome [17] and that the rind of *G. dulcis* (yellow mangosteen) attenuated physiological and metabolic parameters in obese rats through several mechanisms including modulation of gut microbiota [18]. This is anticipated as the rind, peel and seeds of fruits and vegetables often contain high concentrations of bioactive phenolic compounds [2,26]. Hence, fruit waste could be turned into nutraceuticals to generate income for growers with the additional benefit that the use of the peel reduces landfill and harmful environmental impact through reduced biomass decomposition [3].

The phytochemical analysis of *G. mangostana* rind showed the presence of procyanidins, xanthones particularly α-mangostin, anthocyanins and hydroxycitric acid. We measured approximately 140 mg of anthocyanins/100 g of rind which is similar to 179.5 mg of anthocyanin/100 g of rind previously reported [27]. Based on the food intakes in HM rats, we calculated doses of about 168 mg/kg/day, 355 mg/kg/day, 3.9 mg/kg/day and 11.8 mg/kg/day of α-mangostin, procyanidins, anthocyanins and hydroxycitric acid, respectively. Hence, we are proposing that this combination of bioactive compounds in the purple mangosteen rind leads to the range of physiological, metabolic, liver and cardiovascular responses that we report here.

Adipose tissue is an active endocrine organ which secretes cytokines and hormones [28]. The expansion of adipocytes in obesity contributes to chronic low-grade local and systemic inflammation which cause physiological and metabolic abnormalities such as insulin resistance and endothelial dysfunction [29,30]. Further, reduction in adipocyte size was linked to the reduction of white adipose tissue pro-inflammatory cytokines [31,32]. Our study showed that obese rats given *G. mangostana* rind had decreased mean retroperitoneal adipocyte size. This finding was similar to previous findings showing decreased retroperitoneal and epididymal adipocyte size in mice treated with α-mangostin (10–50 mg/kg/day for 5 days–12 weeks) [31,32,33]. This suggests that α-mangostin could ameliorate obesity by reducing the expansion of fat cells. α-Mangostin also reduced ageing-related adipose tissue inflammation by decreasing macrophage content, changing pro-inflammatory macrophage polarisation and reducing pro-inflammatory cytokines and chemokines concentration, specifically IL-1β, iNOS and TNF [32]. The reduced inflammation was partly due to the downregulation of nuclear factor κ-light-chain-enhancer of activated B cells (NF-κB) and mitogen-activated protein kinase (MAPK) pathways in adipose tissue [32]. Moreover, α-mangostin (IC_50_ value 20 µM) prevented adipocyte hypertrophy by its in vitro cytotoxicity against 3T3-L1 cells and fatty acid synthase inhibition as well as by suppressing lipid accumulation in differentiating adipocytes and promoting lipolysis in mature adipocytes [34]. In a similar cell line, α-mangostin inhibited lipid accumulation by inhibition of peroxisome proliferator activated receptor-γ (PPARγ) expression, limited adipocyte differentiation, increased glucose uptake and release of free fatty acids by upregulation of glucose transporter 4 (GLUT4) and leptin [35]. These actions could lead to reduced body weight gain, and epididymal and retroperitoneal fat mass accumulation as shown with administration of 50 mg/kg body weight of α-mangostin in high-fat diet-fed mice [33]. The same dose of α-mangostin for 12 weeks reduced body weight, epididymal fat pads and fatty liver in mice despite unchanged food intake [31].

α-Mangostin improved lipid serum profiles such as reduced cholesterol, triglycerides and free fatty acids [31,32,33]. Moreover, α-mangostin administration at 25, 50 and 100 mg/kg/day in diabetic rats dose-dependently reduced total cholesterol, triglycerides, low-density lipoproteins (LDL) and very low-density lipoproteins (VLDL), while increasing serum high-density lipoproteins (HDL) concentrations [36]. Supplementation of 200 mg/kg/day of α-mangostin in high-fat diet-fed rats reduced overall body weight, liver weight, triglyceride, total cholesterol, free fatty acids and serum glucose concentrations compared to control [37]. The treatment also reduced epididymal fat, mesenteric fat, retroperitoneal fat and inguinal fat compared to control. Protein expression analysis showed α-mangostin increased AMP-activated protein kinase (AMPK) phosphorylation, increased expression of sirtuin 1 (SIRT-1) and reduced PPARγ expression [37]. The decrease in PPARγ suggested decrease of lipogenesis in adipocyte differentiation. However, in our study, only reduction of non-esterified fatty acids was observed with no change in total cholesterol or triglycerides concentrations. This could indicate improved lipolysis from triglycerides due to increased demand for fatty acid oxidation.

The improvement in liver structure was consistent with an improved liver function. Treatment with α-mangostin reduced liver weight [33], which we did not find. However, we found a reduction in liver fat vacuole size which was also noted previously [31,33]. Also, α-mangostin treatment upregulated liver AMPK, SIRT-1 and PPARγ showing that this compound could ameliorate obesity and liver steatosis by these pathways [33]. Additionally, α-mangostin decreased the expression of liver fatty acid synthesis genes such as lipoprotein lipase (LPL), stearoyl-CoA desaturase 1 (SCD-1) and sterol regulatory element-binding protein 1c (SREBP-1c) [31]. Further, α-mangostin decreased both alanine transaminase and aspartate transaminase activities in α-mangostin-treated mice compared to high-fat diet-fed mice [33] and aged mice [32]. Additionally, both α-mangostin and γ-mangostin reduced cellular lipid accumulation and liver function enzymes in HepG2 and L02 cells [38]. Both α-mangostin and γ-mangostin enhanced the expression of SIRT1 and the phosphorylation of liver kinase B1 (LKB1) and AMPK which increased fatty acid oxidation and decreased fatty acid synthesis [38]. However, in our study, only reduction in aspartate transaminase was observed. These results showed that α-mangostin has hepatoprotective properties and is a good candidate compound to reverse liver steatosis. The reduction in infiltration of inflammatory cells and collagen deposition in the liver by mangosteen rind is consistent with previous findings [31,32]. These changes were associated with the reduced pro-inflammatory cytokine genes (TNF, monocyte chemoattractant protein-1 (MCP-1) and C-C chemokine receptor type 2 (CCR2)) together with increased anti-inflammatory cytokine gene, IL-10 [31]. Similarly, oral supplementation of 40 mg/kg α-mangostin reduced inflammatory markers COX-2 and IL-6 in lipopolysaccharide (LPS)-induced mice model [39].

The reduction of liver glycogen in *G. mangostana* rind-treated rats suggested that α-mangostin increased liver glycogen consumption which could be due to increased glycogenolysis or decreased glycogenesis or both due to increased metabolic demand, despite the high-simple carbohydrate supply from high-carbohydrate, high-fat diet. The reduction in liver glycogen could mimic the metabolic changes in fasting. Reduction in liver glycogen stores during fasting triggered the liver-brain-adipose neural axis to increase fatty acid and glycerol release from white adipose tissue [40].

The reduction in glucose response area under the curve in HM rats is consistent with previous studies showing the insulin sensitising effect of α-mangostin in high-fat diet-fed mice due to increased concentration of phosphorylated insulin receptor substrate 1 (IRS-1) and phosphorylated Akt [31]. Supplementation of both 25 and 50 mg/kg/day of α-mangostin also improved insulin resistance through increased phosphorylation of Akt in epididymal adipose tissue of aged rats [32]. In addition, procyanidins improved insulin resistance, by improving glucose uptake and reducing lipogenesis, oxidative stress and inflammation through various pathways [41]. Administration of 500 mg/kg/day of procyanidins in diabetic rats improved plasma glucose concentration and β-cell function. In the same study, doses of 250 mg/kg/day and 500 mg/kg/day of procyanidins improved glucose tolerance [42], which was also observed in this study using approximately 355 mg/kg/day of procyanidins.

The improvement of cardiovascular symptoms was consistent with the decreased infiltration of inflammatory cells in the left ventricular tissue. Supplementation of *G. mangostana* pericarp extract (200 mg/kg/day) showed improvements in blood pressure, improvement in haemodynamic status, decreased left ventricular weight and cardiac wall thickness in nitro-L-arginine methyl ester (L-NAME)-induced cardiovascular remodelling in rats by reduction of inflammation through reduced expression of TNF and iNOS [43]. The administration of 200 mg/kg/day of α-mangostin showed cardioprotective effects in isoproterenol-induced myocardial infarction by preventing the decrease in the activities of endogenous antioxidants (superoxide dismutase, catalase, glutathione peroxidase, glutathione S-transferase and glutathione) and reduced activities of serum marker enzymes such as lactate dehydrogenase, creatine phosphokinase, aspartate transaminase and alanine transaminase [44]. α-Mangostin protected against cardiac reperfusion injury by reducing infarct area, sustaining cardiac mechanical work and preventing the reduction in cardiac ATP and phosphocreatine concentrations in the reperfused rat myocardium by reducing malondialdehyde and 4-hydroxynonenal and increasing reduced glutathione content [45].

Endothelial dysfunction arises from an imbalance in vasodilatory agents such as nitric oxide, prostacyclin and endothelial-derived hyperpolarising factors and vasoconstricting agents including angiotensin-II, prostaglandin and endothelin-1 [30]. Macrophage infiltration of the adipose tissue is the main contributor of inflammation linked to endothelial dysfunction. In obese mice, macrophage-specific genes such as MCP-1, macrophage inflammatory protein-1α, CD11b, CD68 and F4/80 ratio were up-regulated [46]. Decreased infiltration of inflammatory cells may explain the improvement in relaxation and contraction responses of aortic ring experiments in treated rats. Noradrenaline-induced thoracic aortic contraction responses were increased in HM rats compared to H rats and acetylcholine-induced relaxation was increased in HM rats compared to H rats. This suggests that the phytochemicals in the *G. mangostana* rind limit the endothelial changes caused by high-carbohydrate, high-fat diet. Previously, *G. mangostana* rind extract increased endothelial function in the aorta of obese rats by reducing endothelial vasoconstriction through increased nitric oxide production and decreased reactive oxygen species generation [47]. This study found that xanthone-rich fraction was less effective in producing vasorelaxation effects compared to fraction containing epicatechin which is a constituent of procyanidins [47]. Our previous study showed that the intake of 40 mg/kg/day of procyanidins, lower than the current dose, improved cardiovascular structure and function in high-carbohydrate, high-fat diet-fed rats [17]. Procyanidin showed cardioprotective effects by promoting blood vessel relaxation and inhibiting LDL oxidation [48] as well as acting as antioxidant and anti-inflammatory agents [49]. Further, a prospective cohort study showed that *G. mangostana* extract supplementation in diabetic individuals improved endothelial dysfunction by increasing endothelial progenitor cells and the antioxidant enzyme superoxide dismutase, along with decreased concentrations of inflammatory and oxidative stress markers such as IL-1, IL-6, TNF, high-sensitivity C-reactive protein (hs-CRP), malondialdehyde and nitric oxide [50].

In this study, we did not find any increase in faecal lipid excretion despite previous studies showing high in vitro activity of α-mangostin as a pancreatic lipase inhibitor [51]. This indicates that the bioactive compounds from *G. mangostana* did not alter gut lipid absorption in vivo and suggests that the mechanisms of weight loss in HM rats could involve increased oxidation of fatty acids. This can be further supported by the decrease in feed efficiency along with reduction in body weight and whole-body fat mass.

Toxicity is a potential problem with chronic dosage of natural products. However, purple mangosteen pericarp powder at a single dose of 3g/kg in rats caused no alterations in body or organ weights or cytoarchitecture with mild changes in haematological parameters [52]. A chronic toxicity study of *G. mangostana* pericarp extract (24.4% α-mangostin) at a dose of 10–1000 mg/kg/day in Wistar rats, so much higher than the dose in HM rats of 168 mg/kg/day, did not show any overt pharmacological signs and haematological abnormalities but the highest dose was suggested to affect the liver [53]. However, we did not find any liver abnormalities in HM rats. The doses of procyanidins, anthocyanins and hydroxycitric acid in HM rats are lower than doses causing minimal or no toxicity in rats. The dose of procyanidin in HM rats of 355 mg/kg/day is much less than the acute dose of 2000 mg/kg causing no micronucleated erythrocytes or an acute LD50 of greater than 5000 mg/kg in rats [54]. Our previous studies with the anthocyanin, cyanidin glucoside, in the same model of diet-induced metabolic syndrome showed improvements in organ structure and function at doses of 8 mg/kg/day for 8 weeks with no toxicity [55]. No toxicity to anthocyanins was reported at doses of 20 mg/kg/day in rats [56]. Possible toxicity in patients is an important consideration if our findings in rats are to be used as a basis for therapeutic intervention. However, limited studies in humans have not reported toxicity of the compounds present in purple mangosteen rind. The dose of α-mangostin in HM rats of 168 mg/kg/day is equivalent to about 1680 mg/day for an adult human [50]. Single doses of a lower dose of α-mangostin (305 mg [57] or 330 mg [58]) to healthy humans showed no gastrointestinal distress or other symptoms. Further, no human toxicity has been shown to procyanidins, anthocyanins [59] or hydroxycitric acid [60].

The rind did not change food and water intake, but the total energy intake was lower in treatment rats than control rats. This explained the reduction in body weight gain and feed efficiency. The reduction of fat mass but not lean mass in HM rats showed that *G. mangostana* treatment did not affect muscle mass or muscle protein degradation. Furthermore, the interactions between different constituents present in the rind such as xanthones, anthocyanins, hydroxycitric acid and procyanidins could have additive or synergistic effects in producing the observed effects. We previously suggested that the interactions of procyanidins and citric acid in *G. humilis* rind improved cardiovascular parameters in diet-induced metabolic syndrome rats [17]. Additionally, we have shown that supplementation of 8 mg/kg/day of cyanidin 3-glucoside improved parameters associated with metabolic syndrome in this rat model [55]. Furthermore, previous studies showed phytochemical combinations including anthocyanins in *G. indica* rind showed antioxidant and hepatoprotective effects in rats [61]. We also noted the presence of hydroxycitric acid in the rind which contributed to the intake of 11.8 mg/kg/day of hydroxycitric acid in HM rats. The hydroxycitric acid content in the *G. mangostana* rind is considerably lower than the quantity found in *G. gummi-gutta* (*G. cambogia*) which amounts to about 50–60% *w*/*w* [62]. Previous studies showed that hydroxycitric acid has anti-obesity properties at 710–2390 mg/kg/day, much higher than the dose given in this study [62,63,64]. Thus, while α-mangostin is the most likely bioactive compound, the presence of these other compounds could enhance the effects of α-mangostin.

## 5. Conclusions

This study showed *G. mangostana* rind improved physiological, metabolic, liver and cardiovascular symptoms in rats with diet-induced metabolic syndrome, probably initiated by decreased infiltration of inflammatory cells into the heart, liver and other organs. These biological effects were most likely due to additive or synergistic responses of compounds in the rind including α-mangostin, procyanidins, hydroxycitric acid and anthocyanins. These findings support the role of *G. mangostana* rind to attenuate metabolic syndrome, indicating a more extensive role for this portion of a widespread tropical fruit, now mostly discarded as a waste product, as a functional food.

## Figures and Tables

**Table 1:** Effects of *Garcinia mangostana* rind on metabolic and physiological parameters.

Variables	C	CM	H	HM	*p* Value		
					Diet	Treatment	Diet × Treatment
Physiological parameters							
Initial body weight, g	338 ± 1 ^a^	339 ± 1 ^a^	337 ± 1 ^a^	337 ± 1 ^a^	0.21	0.41	0.39
Body weight at 8 weeks, g	358 ± 7 ^b^	371 ± 5 ^b^	425 ± 7 ^a^	416 ± 6 ^a^	<0.0001	0.76	0.08
Body weight at 16 weeks, g	407 ± 8 ^b^	346 ± 7 ^c^	515 ± 13 ^a^	438 ± 12 ^b^	<0.0001	<0.0001	0.44
Food intake, g/day	41.0 ± 2.1 ^a^	36.3 ± 2.3 ^a^	27.5 ± 2.9 ^b^	24.3 ± 1.9 ^b^	<0.0001	0.10	0.75
Water intake, g/day	24.6 ± 2.6 ^a^	25.6 ± 2.5 ^a^	28.4 ± 3.4 ^a^	32.3 ± 3.8 ^a^	0.09	0.44	0.64
α-mangostin intake, mg/kg/day	-	280 ± 4	-	168 ± 10	-	-	-
Procyanidin intake, mg/kg/day	-	653 ± 8	-	355 ± 6	-	-	-
Anthocyanin intake, mg/kg/day	-	7.3 ± 0.09	-	3.9 ± 0.06	-	-	-
Hydroxycitric acid intake, mg/kg/day	-	21.8 ± 0.28	-	11.8 ± 0.18	-	-	-
Energy intake, kJ/day	460 ± 26 ^bc^	401 ± 28 ^c^	605 ± 48 ^a^	543 ± 39 ^ab^	0.0003	0.10	0.96
Feed efficiency, g/kJ	0.11 ± 0.01 ^b^	−0.06 ± 0.01 ^d^	0.15 ± 0.02 ^a^	0.02 ± 0.01 ^c^	<0.0001	<0.0001	0.25
Body weight gain (8–16 weeks), %	13.7 ± 0.9 ^b^	−6.7 ± 0.8 ^d^	21.4 ± 2.8 ^a^	3.0 ± 1.9 ^c^	<0.0001	<0.0001	0.57
Abdominal circumference at 8 weeks, cm	16.1 ± 0.2 ^b^	16.6 ± 0.2 ^b^	20.3 ± 0.6 ^a^	20.2 ± 0.5 ^a^	<0.0001	0.62	0.53
Abdominal circumference at 16 weeks, cm	18.5 ± 0.4 ^b^	16.2 ± 0.2 ^c^	22.6 ± 0.3 ^a^	17.9 ± 0.3 ^b^	<0.0001	<0.0001	0.0003
Whole-body lean mass at 8 weeks, g	291 ± 9 ^a^	302 ± 6 ^a^	308 ± 4 ^a^	310 ± 5 ^a^	0.06	0.33	0.59
Whole-body lean mass at 16 weeks, g	293 ± 11 ^ab^	283 ± 4 ^bc^	297 ± 7 ^ab^	311 ± 5 ^a^	0.031	0.79	0.10
Whole-body fat mass at 8 weeks, g	55 ± 9 ^b^	50 ± 4 ^b^	96 ± 8 ^a^	84 ± 6 ^a^	<0.0001	0.26	0.69
Whole-body fat mass at 16 weeks, g	95 ± 9 ^b^	42 ± 3 ^c^	211 ± 13 ^a^	102 ± 8 ^b^	<0.0001	<0.0001	0.004
Bone mineral content at 8 weeks, g	11.0 ± 0.4 ^b^	10.1 ± 0.2 ^b^	12.4 ± 0.3 ^a^	11.9 ± 0.2 ^a^	<0.0001	0.053	0.62
Bone mineral content at 16 weeks, g	10.9 ± 0.3 ^c^	12.4 ± 0.4 ^b^	16.4 ± 0.5 ^a^	12.5 ± 0.4 ^b^	<0.0001	0.015	<0.0001
Bone mineral density at 8 weeks, g/cm^2^	0.160 ± 0.003 ^a^	0.158 ± 0.003 ^a^	0.170 ± 0.002 ^a^	0.163 ± 0.004 ^a^	0.026	0.17	0.52
Bone mineral density at 16 weeks, g/cm^2^	0.175 ± 0.003 ^b^	0.162 ± 0.003 ^c^	0.185 ± 0.003 ^a^	0.166 ± 0.003 ^bc^	0.029	<0.0001	0.32
Body mass index at 16 weeks, g/cm^2^	0.63 ± 0.01 ^bc^	0.60 ± 0.01 ^c^	0.78 ± 0.02 ^a^	0.67 ± 0.02 ^b^	<0.0001	<0.0001	0.015
Retroperitoneal fat, mg/mm tibial length	250 ± 21 ^b^	109 ± 8 ^c^	516 ± 35 ^a^	258 ± 25 ^b^	<0.0001	<0.0001	0.020
Epididymal fat, mg/mm tibial length	97 ± 16 ^b^	59± 8 ^c^	169 ± 17 ^a^	88 ± 10 ^b^	0.0003	<0.0001	0.10
Omental fat, mg/mm tibial length	156 ± 13 ^b^	76 ± 4 ^c^	267 ± 13 ^a^	145 ± 11 ^b^	<0.0001	<0.0001	0.051
Total abdominal fat, mg/mm tibial length	502 ± 43 ^b^	244 ± 12 ^c^	952 ± 54 ^a^	492 ± 39 ^b^	<0.0001	<0.0001	0.014
Visceral adiposity index, %	5.72 ± 0.40 ^b^	3.23 ± 0.16 ^d^	8.79 ± 0.38 ^a^	5.40 ± 0.34 ^b^	<0.0001	<0.0001	0.18
Heat production, 16 week, kcal/h	3.98 ± 0.31 ^a^	2.66 ± 0.24 ^b^	4.48 ± 0.24 ^a^	3.61 ± 0.23 ^a^	0.015	0.001	0.40
Heat production area under the curve	2864 ± 151 ^ab^	1919 ± 149 ^c^	3235 ± 80 ^a^	2606± 111 ^b^	<0.0001	<0.0001	0.08
RER, 16 week	0.984 ± 0.02 ^a^	0.958 ± 0.01 ^a^	0.852 ± 0.02 ^b^	0.869 ± 0.02 ^b^	<0.0001	0.98	0.37
RER area under the curve, 16 week	708 ± 10 ^a^	696 ± 6 ^a^	614 ± 7 ^b^	626 ± 7 ^b^	<0.0001	0.79	0.64
Mean liver fat vacuole area, µm^2^	14.1 ± 0.7 ^d^	37.4 ± 2.3 ^c^	142.1 ± 13.8 ^a^	59.9 ± 4.4 ^b^	<0.0001	<0.0001	<0.0001
Mean retroperitoneal adipocyte area, µm^2^	4333 ± 109 ^c^	2935 ± 213 ^d^	9587 ± 482 ^a^	5173 ± 487 ^b^	<0.0001	<0.0001	<0.0001
Faecal lipid content, mg/g	20.8 ± 1.5 ^b^	16.5 ± 0.6 ^b^	40.6 ± 1.5 ^a^	43.4 ± 2.8 ^a^	<0.0001	0.66	0.048
Plasma biochemistry							
Alanine transaminase activity, U/L	28.9 ± 3.0 ^c^	44.1 ± 3.5 ^b^	42.7 ± 5.0 ^b^	62.2 ± 6.0 ^a^	0.002	0.0006	0.64
Aspartate transaminase activity, U/L	86 ± 8 ^b^	87 ± 4 ^b^	177 ± 22 ^a^	112 ± 12 ^b^	0.002	0.07	0.06
Total cholesterol, mmol/L	1.49 ± 0.06 ^b^	0.89 ± 0.07 ^c^	1.69 ± 0.09 ^a^	1.44 ± 0.07 ^a^	<0.0001	<0.0001	0.038
Triglycerides, mmol/L	0.47 ± 0.05 ^b^	0.56 ± 0.10 ^b^	1.15 ± 0.15 ^a^	1.23 ± 0.26 ^a^	<0.0001	0.55	0.97
Non-esterified fatty acids, mmol/L	0.87 ± 0.16 ^c^	1.40 ± 0.25 ^c^	3.27 ± 0.16 ^a^	2.36 ± 0.29 ^b^	<0.0001	0.39	0.002
Basal blood glucose at 8 weeks, mmol/L	2.4 ± 0.1 ^b^	2.5 ± 0.2 ^b^	3.1 ± 0.1 ^a^	3.0 ± 0.1 ^a^	<0.0001	1.00	0.33
Basal blood glucose at 16 weeks, mmol/L	2.7 ± 0.1 ^ab^	2.5 ± 0.2 ^b^	3.0 ± 0.1 ^a^	2.6 ± 0.1 ^ab^	0.024	0.08	0.56
Blood glucose area under the curve at 8 weeks, mmol/L×minutes	495 ± 11 ^b^	483 ± 10 ^b^	580 ± 9 ^a^	572 ± 9 ^a^	<0.0001	0.32	0.84
Blood glucose area under the curve at 16 weeks, mmol/L×minutes	471 ± 17 ^c^	461 ± 13 ^c^	611 ± 33 ^a^	546 ± 15 ^b^	<0.0001	0.016	0.15
Liver wet weight, mg/mm tibial length	234 ± 8 ^b^	246 ± 7 ^b^	355 ± 12 ^a^	358 ± 9 ^a^	<0.0001	0.44	0.61
Liver glycogen, mg/g	12.42 ± 0.60 ^a^	10.82 ± 1.58 ^a^	13.58 ± 0.51 ^a^	4.76 ± 0.57 ^b^	0.007	<0.0001	0.0002
Catalase activity, kU/L	39.1 ± 3.7 ^b^	26.6 ± 4.7 ^b^	55.2 ± 5.8 ^a^	42.4 ± 4.7 ^ab^	0.033	0.008	0.98
C-reactive protein, µg/mL	432 ± 12 ^b^	360 ± 19 ^c^	508 ± 8 ^a^	295 ± 12 ^d^	<0.0001	<0.0001	<0.0001
Cardiovascular parameters							
Systolic blood pressure at 8 weeks, mmHg	115 ± 3 ^b^	117 ± 2 ^b^	136 ± 4 ^a^	132 ± 3 ^a^	<0.0001	0.69	0.37
Systolic blood pressure at 16 weeks, mmHg	117 ± 2 ^b^	105 ± 5 ^b^	131 ± 2 ^a^	109 ± 5 ^b^	0.020	<0.0001	0.16
Left ventricle + septum wet weight, mg/mm tibial length	23.1 ± 1.3 ^a^	19.6 ± 0.8 ^b^	23.2 ± 1.2 ^a^	21.2 ± 0.6 ^ab^	0.41	0.006	0.38
Right ventricle, mg/mm tibial length	4.04 ± 0.18 ^ab^	3.67 ± 0.14 ^b^	4.61 ± 0.17 ^a^	4.05 ± 0.15 ^ab^	0.006	0.006	0.56
Diastolic stiffness constant (*κ*)	21.1 ± 0.4 ^bc^	20.4 ± 0.3 ^c^	26.8 ± 0.8 ^a^	22.2 ± 0.9 ^b^	<0.0001	0.0002	0.0002

Values are expressed as mean ± SEM; n = 8–12. Means with different superscripts (a, b, c, d) differ, *p* < 0.05. C, corn starch diet-fed rats; CM, corn starch diet-fed rats supplemented with *Garcinia mangostana* rind; H, high-carbohydrate, high-fat diet-fed rats; HM, high-carbohydrate, high-fat diet-fed rats supplemented with *Garcinia mangostana* rind; RER, respiratory exchange ratio.

## Data Availability

The data presented in this study are available on request from the corresponding author.

## Supplementary Materials

The following are available online at https://www.mdpi.com/2072-6643/13/2/319/s1.
