# Supplementary material for: Rind from Purple Mangosteen (Garcinia mangostana) Attenuates Diet-Induced Physiological and Metabolic Changes in Obese Rats
Source: Nutrients. 2021 Jan 22;13(2):319. doi: 10.3390/nu13020319 (PMC7912346; doi:10.3390/nu13020319)
Supplement: Supplementary file 1 [file nutrients-13-00319-s001.pdf]

A. HPLC-MS Profile of *G. mangostana* rind

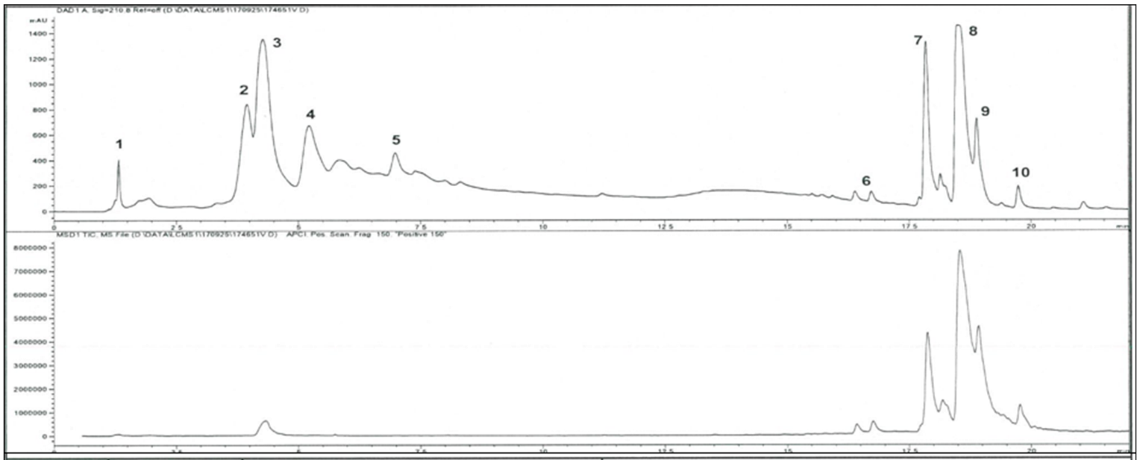

Peak #	RT (min)	Fragment ions [M+H]	Tentative ID (MW)
1	1.3	136, 216, 234, 248, 262, 276	mixed peak (anthocyanins, amino acids, amines)
2	3.9	579	procyanidin (procyanidin B1)
3	4.3	291	flavan-3-ol (epicatechin)
4	5.3	247, 291, 369, 563	flavanol derivative (procyanidin)
5	5.2	247, 369, 563	flavanol derivative (procyanidin)
6	16.4, 16.7	341, 397, 355, 411	xanthone derivatives
7	17.8	341, 397	xanthone derivative (γ-mangostin)
8	18.5	355, 411	xanthone derivtaive (α-mangostin)
9	18.9	465	xanthone derivative (garcinone E)
10	19.8	369, 425	xanthone derivative (β-mangostin)

COMMENTS

The HPLC-MS profile of the test sample Purple Mangosteen Rind is given above with some major components from the plant extracts indicated. The peaks identified are anthocyanin, a range of flavan-3-ols and procyanidin derivatives with xanthones after 15 minutes, based on their characteristic UV-Vis and or MS spectra and literature reporting on constituents identified from *Garcinia* spp. Spectral data in support of peak identification is attached.

B. Spectral Data for Figure 1.

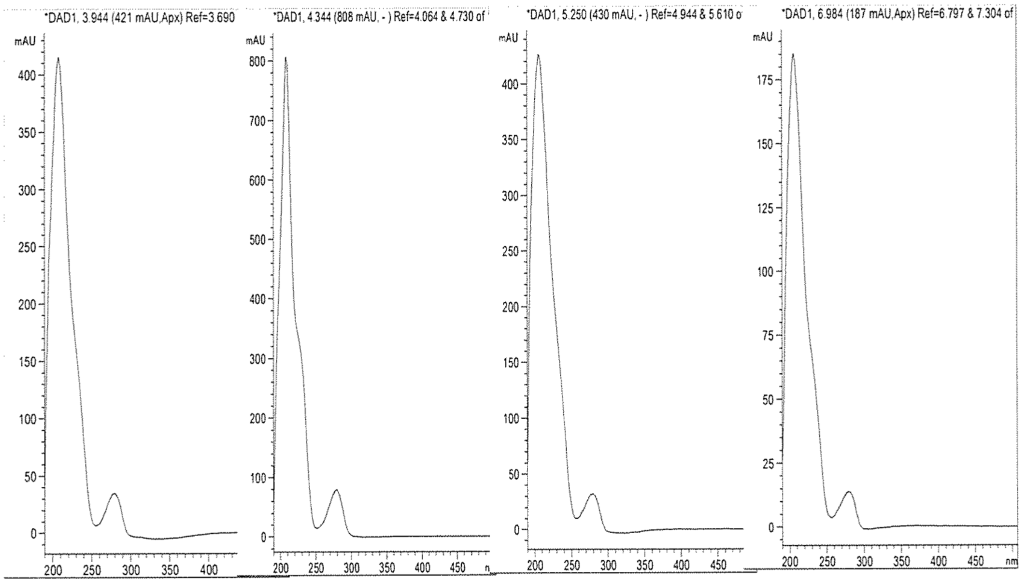

Figure 1. UV-Vis spectra of peaks 2, 3, 4 and 5, left to right, identified as flavan-3-ol derivtaives and procyanidins, with major MS fragment ions of m/z 291, 579 and 563

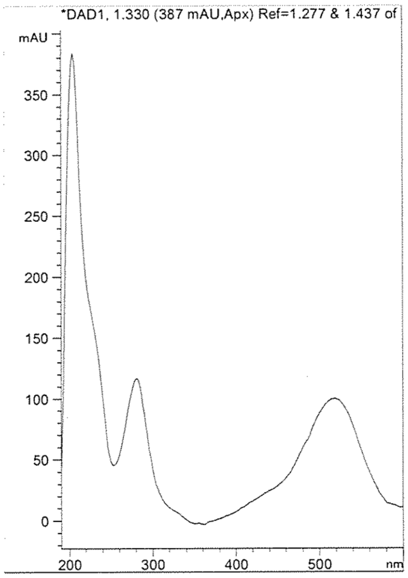

Figure 2. UV-Vis spectra of peak 1 identified as anthocyanin based on common and characterisitic spectra with absorption maxima ~520nm

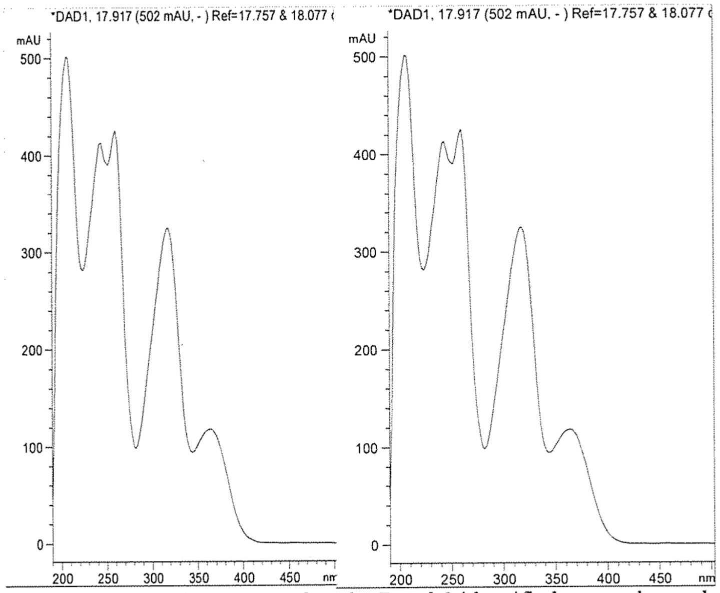

Figure 3. UV-Vis spectra of peaks 7 and 8 identified as xanthone derivatives γ-mangostin and α-mangostin repectively based on characterisitic spectra, MS fragment ions (m/z 397 and 411) and retention time

1. Xanthone and α-mangostin calculation

```
Data File W:\SCPS\ANALYTICAL\LABDATA\LCMS-2\LCMS-2\DATA\171016\174651.D
Sample Name: Garcinia

=====================================================================
Acq. Operator   : JS                              Seq. Line :   3
Acq. Instrument : LCMS-2                           Location : Vial 23
Injection Date  : 16/10/2017 4:55:10 PM                 Inj :   1
                                                 Inj Volume : 0.100 µl
Acq. Method     : D:\LCMS-2\METHODS\QAXANTHONE.M
Last changed    : 16/10/2017 3:54:51 PM by JS
Analysis Method : C:\CHEMSTATION\LCMS\1\METHODS\QA-GARCINIA.M
Last changed    : 19/10/2017 10:07:22 AM
                  (modified after loading)
```

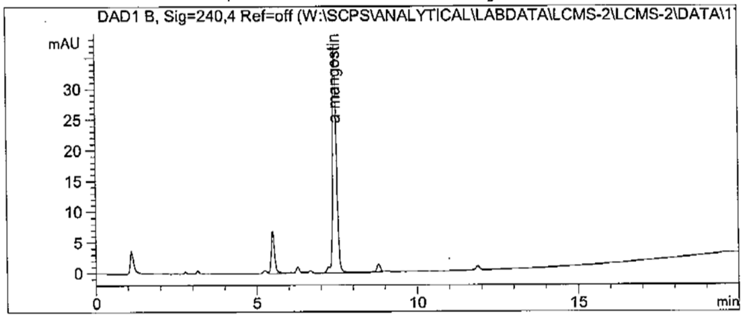


```
=====================================================================
                     ESTD Percent Report
=====================================================================

Sorted By             :      Signal
Calib. Data Modified  :      Thursday, 19 October 2017 10:07:22 AM
Multiplier:                   :     50.0000
Dilution:                     :     25.0000
Sample Amount:               :   212.40000  [ng/ul]
Use Multiplier & Dilution Factor with ISTDs

Signal 1: DAD1 B, Sig=240,4 Ref=off
Uncalibrated Peaks    :      using compound a-mangostin
```

RetTime [min]	Type	Area [mAU*s]	Amt/Area	Amount %	Grp	Name
5.502	VB	47.91166	3.19564e-5	0.901061		?
7.443	BB	286.35910	3.21434e-5	5.416992		a-mangostin
8.791	BB	9.38179	3.19564e-5	0.176441		?
15.157		-	-	-		garcinol

Totals : 6.494493

1 Warnings or Errors :

Warning : Calibrated compound(s) not found

===

*** End of Report ***

2. Procyanidin calculation

```
Data File D:\DATA\200205\2020-02-05 12-01-05\174651.D
Sample Name: Garcinia

=====================================================================
Acq. Operator   : ASK                           Seq. Line :   2
Acq. Instrument : CHROMO-6                       Location : Vial 11
Injection Date  : 5/02/2020 12:24:06 PM               Inj :   1
                                               Inj Volume : 10.000 µl
Different Inj Volume from Sequence !    Actual Inj Volume : 20.000 µl
Acq. Method     : D:\DATA\200205\2020-02-05 12-01-05\USPPROCYA.M
Last changed    : 7/03/2019 12:17:08 PM by k shepherd
Analysis Method : D:\METHODS\USPPROCYA.M
Last changed    : 6/02/2020 10:23:31 AM by TC
                  (modified after loading)
Method Info     : USP procyanidin SEC column 300 mm (D) THF 1.0mL/min  280nm 10uL 25C
```

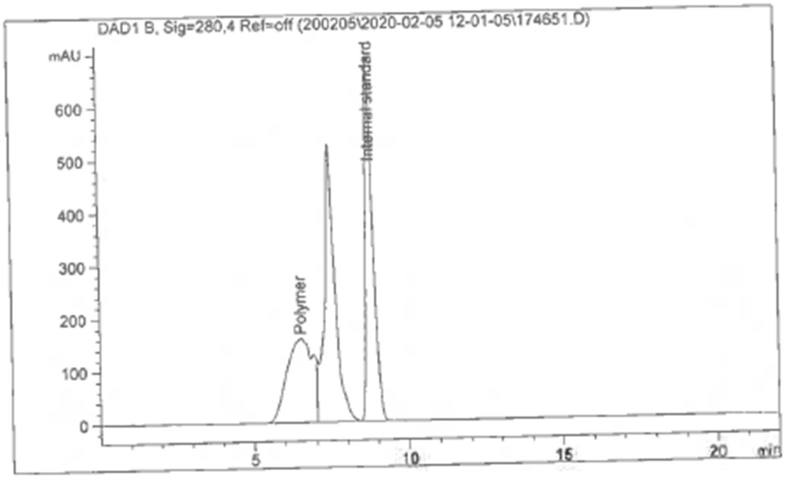


```
=====================================================================
                       Area Percent Report
=====================================================================

Sorted By             :      Signal
Calib. Data Modified  :      6/02/2020 10:23:30 AM
Multiplier:                   :       1.0000
Dilution:                     :       1.0000
Do not use Multiplier & Dilution Factor with ISTDs


Signal 1: DAD1 B, Sig=280,4 Ref=off

Peak RetTime  Type   Width      Area      Area    Name
  #   [min]          [min]    [mAU*s]       %
----|-------|------|-------|----------|--------|---------------------
   1   6.521 BV     0.8966 9097.04492  27.4748 Polymer
   2   7.279        0.0000    0.00000   0.0000 Monomers
   3   8.783 VBA    0.2774 1.13735e4   34.3502 Internal standard

Totals :                   2.04706e4   61.8250


1 Warnings or Errors :

Warning : Calibrated compound(s) not found

=====================================================================
                        *** End of Report ***
```

3. Hydroxycitric acid calculation

Data File D:\DATA\200213\2020-02-13 14-15-17\174651.D
Sample Name: Garcinia rind

Acq. Operator	: AW	Seq. Line :	6
Acq. Instrument	: CHROMO-6	Location :	Vial 64
Injection Date	: 13/02/2020 4:05:15 PM	Inj :	1
		Inj Volume :	20.000 µl

Acq. Method : D:\DATA\200213\2020-02-13 14-15-17\USPHCA.M
Last changed : 9/01/2019 3:04:07 PM by kms
Analysis Method : D:\METHODS\ARCHIVE2018\USPHCA.M
Last changed : 21/02/2020 2:12:09 PM by TC
(modified after loading)
Method Info : C18,4u; 250 x 4.6mm; 1mL/min K Phosphate pH 2.5, 215nm

Additional Info : Peak(s) manually integrated

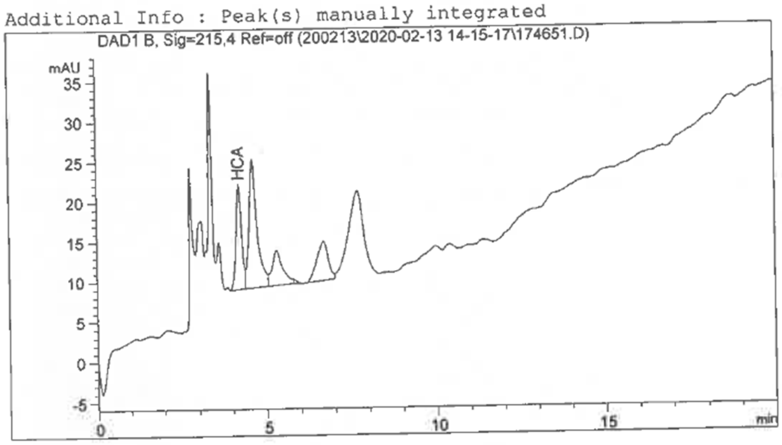

ESTD Percent Report

Sorted By : Signal
Calib. Data Modified : 21/02/2020 2:10:07 PM
Multiplier: : 1.0000
Dilution: : 10.0000
Sample Amount: : 214.00000 [mg/ml]
Use Multiplier & Dilution Factor with ISTDs

Signal 1: DAD1 B, Sig=215,4 Ref=off

RetTime [min]	Type	Area [mAU*s]	Amt/Area	Amount %	Grp	Name
3.572		-	-	-		HCA lactone
4.208	BV	146.17972	8.54518e-4	0.583707		HCA
7.540		-	-	-		citric acid

Totals : 0.583707

1 Warnings or Errors :

Warning : Calibrated compound(s) not found

*** End of Report ***

4. Anthocyanin Calculation

Data File D:\DATA\200204\174651D.D　　　　　　　　　　　　　　Sample Name: Garcinia

===

Injection Date　: 2/5/2020 11:01:59 PM　　　　　Seq. Line : 12
Sample Name　　: Garcinia　　　　　　　　　　　Location : Vial 10
Acq. Operator　: ASK　　　　　　　　　　　　　　　　Inj : 1
　　　　　　　　　　　　　　　　　　　　　　　Inj Volume : 10 µl
Different Inj Volume from Sequence !　　Actual Inj Volume : 20 µl
Acq. Method　　: D:\METHODS\BPBILLB.M
Last changed　　: 2/5/2020 11:02:09 PM by ASK
　　　　　　　　(modified after loading)
Analysis Method : D:\METHODS\ANBILLB.M
Last changed　　: 2/6/2020 3:58:46 PM by ASK
　　　　　　　　(modified after loading)

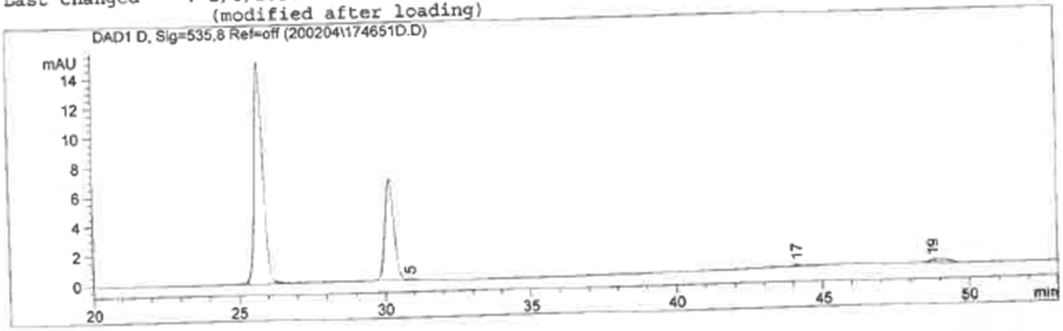

===

Area Percent Report

===

Sorted By　　　　　　　:　　Signal
Calib. Data Modified　:　　3/14/2019 1:41:48 PM
Multiplier　　　　　　:　　1.0000
Dilution　　　　　　　:　　125.0000
Sample Amount　　　　:　　130.50000 [mg]　(not used in calc.)
Use Multiplier & Dilution Factor with ISTDs

Signal 1: DAD1 D, Sig=535,8 Ref=off

Peak #	RetTime [min]	Type	Width [min]	Area [mAU*s]	Area %	Name
1	21.943		0.0000	0.00000	0.0000	1
2	24.961		0.0000	0.00000	0.0000	2
3	25.732	BB	0.3100	301.56213	8.200e3	?
4	26.991		0.0000	0.00000	0.0000	3
5	28.381		0.0000	0.00000	0.0000	4
6	30.174	BV	0.3211	142.84793	3.884e3	?
7	30.857	VB	0.3492	3.12091	84.8587	5
8	33.020		0.0000	0.00000	0.0000	6
9	33.767		0.0000	0.00000	0.0000	7
10	36.489		0.0000	0.00000	0.0000	8
11	38.699		0.0000	0.00000	0.0000	10
12	39.483		0.0000	0.00000	0.0000	9
13	40.085		0.0000	0.00000	0.0000	11
14	41.391		0.0000	0.00000	0.0000	12
15	42.035		0.0000	0.00000	0.0000	13
16	42.824		0.0000	0.00000	0.0000	14
17	42.965		0.0000	0.00000	0.0000	15
18	44.107	BP	0.1487	1.28195	34.8566	17
19	44.519		0.0000	0.00000	0.0000	16
20	45.878		0.0000	0.00000	0.0000	18
21	48.771	BV F	0.1750	2.53140	68.8296	19
22	48.905	VP	0.4572	8.37753	227.7881	?
23	49.281		0.0000	0.00000	0.0000	20
Totals :				459.72185		

Results obtained with enhanced integrator!
2 Warnings or Errors :
